# Myocarditis incidence and hospital mortality from 2007 to 2022: insights from a nationwide registry

**DOI:** 10.1007/s00392-024-02494-3

**Published:** 2024-08-26

**Authors:** Felix A. Rottmann, Christian Glück, Klaus Kaier, Xavier Bemtgen, Alexander Supady, Constantin von zur Mühlen, Dirk Westermann, Tobias Wengenmayer, Dawid L. Staudacher

**Affiliations:** 1 Department of Medicine IV Nephrology and Primary Care, Medical Center, Faculty of Medicine, University of Freiburg, Freiburg, Germany; 2 Interdisciplinary Medical Intensive Care, Medical Center, Faculty of Medicine, University of Freiburg, Freiburg, Germany; 3 Institute for Medical Biometry and Statistics, Faculty of Medicine, University of Freiburg, Freiburg, Germany; 4 Department of Cardiology, Pneumology, Angiology and Intensive Care, Ortenau Clinical Center Offenburg-Kehl, Offenburg, Germany; 5 Department of Cardiology and Angiology, Heart Center Freiburg University, Faculty of Medicine, University of Freiburg, Freiburg, Germany

**Keywords:** Myocarditis, COVID-19, Survival, National registry, Hospitalization

## Abstract

**Objectives:**

To investigate the burden of disease of myocarditis in Germany and identify similarities and differences in myocarditis with or without COVID-19.

**Methods:**

All patients hospitalized with myocarditis in Germany were included in this nationwide retrospective analysis. Data were retrieved from the Federal Statistical Office of Germany (DESTATIS) for the years from 2007 to 2022. The primary endpoint was hospital mortality.

**Results:**

A total of 88,159 patients hospitalized with myocarditis were analyzed. Annual cases increased from 5100 in 2007 to 6593 in 2022 (*p* < 0.001 for trend) with higher incidence during winter months. Incidence per 100,000 inhabitants was 6.2 in 2007 rising to 7.8 in 2022 (*p* < 0.001 for trend). Hospital mortality remained constant at an average of 2.44% (*p* = 0.164 for trend). From 2020 to 2022, 1547/16,229 (9.53%) patients were hospitalized with both, myocarditis and COVID-19 (incidence 0.62/100,000 inhabitants and 180/100,000 hospitalizations with COVID-19). These patients differed significantly in most patient characteristics and had a higher rate of hospital mortality compared to myocarditis without COVID-19 (12.54% vs. 2.26%, respectively, *p* < 0.001).

**Conclusions:**

Myocarditis hospitalizations were slowly rising over the past 16 years with hospital mortality remaining unchanged. Incidence of hospitalizations with combined myocarditis and COVID-19 was low, but hospital mortality was high.

**Supplementary Information:**

The online version contains supplementary material available at 10.1007/s00392-024-02494-3.

## Introduction

Incidence of myocarditis is estimated at around 1.8 million cases per year worldwide with incidences varying widely between 1 and > 10 cases/100.000 people/year [1–6]. Globally, the age-standardized incidence rate decreased over the last 30 years, while mortality remained unchanged [1]. Clinically, differentiation between myocarditis and other cardiac diseases, including coronary artery disease, can be difficult, considering similar symptoms, electrocardiography patterns, and echocardiographic findings [7]. To some extent, this may explain the varying incidences reported previously. The gold standard for confirmation of myocarditis is myocardial biopsy [8]. Current guidelines, however, suggest myocarditis to be diagnosed alternatively by magnetic resonance imaging (MRI) and elevated troponin levels, not requiring myocardial biopsy [9–12].

Most myocarditis cases in high-income countries are associated with viral agents like influenza and coronaviruses including SARS-CoV-2 [7, 13]. Of note, some cardiotropic viruses, such as Parvovirus B19 and HHV-6 (human herpesvirus 6), are not only found in myocardial biopsies in myocarditis but also in healthy individuals as “innocent bystanders” [14, 15]. Infectious agents further include bacteria, protozoa, and fungi, while systemic immune-mediated diseases (e.g., sarcoidosis) [7, 13, 16] and side effects of checkpoint inhibitors [17] or vaccinations [18] represent other rare causes. SARS-CoV-2 vaccination coincides with myocarditis only in a minority of people [18].

Ammirati et al. reported an incidence of probable or confirmed myocarditis in 240/100,000 patients hospitalized for coronavirus disease 2019 (COVID-19) and a fulminant presentation in 39% of these cases [19]. These findings are in line with similar data published during the COVID-19 pandemic in Germany [20].

In myocarditis associated with COVID-19, both direct viral tissue damage as well as a dysregulated immune response are likely to injure the myocardium [21]. Other mechanisms of tissue damage are endothelialitis and microvascular thrombosis, systemic hyperinflammation, and reduced oxygen supply [22].

Clinical courses of myocarditis include a wide range from asymptomatic inflammation with full recovery to severe courses characterized by acute cardiac failure or cardiogenic shock, severe arrhythmia, or sudden death. Some patients develop chronic cardiomyopathy with mild or severe reduced left ventricular function [7, 10]. Treatment of the underlying cause (if detected) should be combined with standard heart failure medication. Immunosuppression is not generally advised [12, 23].

The goal of our current analysis was to investigate the burden of disease of myocarditis in Germany including changes since 2020 during the COVID-19 pandemic.

## Methods

### Data source

Data for this research were derived from the Federal Statistical Office of Germany (German: Statistisches Bundesamt, short: DESTATIS). By German law, all reimbursement-related healthcare patient data (e.g., diagnosis-related group, DRG) are reported to DESTATIS. These data, including OPS (“Operationen- und Prozedurenschlüssel”, English: Operation and Procedure Classification System) codes used to classify various tests and treatments and ICD (International Statistical Classification of Diseases and Related Health Problems) codes, can be obtained from DESTATIS in an anonymous data package. DESTATIS blanks some data requests for small subgroups due to the potential impact of fine data granulation on anonymity. No ethics approval is needed to access and analyze anonymized DESTATIS data. Statistics on COVID-19 were derived from the Robert Koch Institute and are openly available from 2020, week 10 until 2023, week 22.

### Study population

We included all patients from the DESTATIS database diagnosed with myocarditis from January 1, 2007, to December 31, 2022. Myocarditis could be coded as the main or secondary diagnosis. Cases were identified using specific ICD codes I40* (coding for acute myocarditis). There were no exclusion criteria.

### Outcome measures

The primary outcome was the incidence of hospitalization with myocarditis. Secondary outcomes were hospital survival (or in-hospital mortality, as applicable), the need for mechanical circulatory support (MCS), mechanical ventilation, and length of hospital stay.

### Predefined subgroups

As a predefined subgroup, the incidence of myocarditis in the context of COVID-19 (i.e., myocarditis with COVID-19) was investigated using the ICD codes (U07.1!, U07.2!, U07.3!, or U07.4!). Patients with COVID-19 were only included in this research when also coded for myocarditis.

### Other patient characteristics

Myocardial biopsy and MCS were identified using the following OPS codes: myocardial biopsy (1–497.1 and 1–497.2), intra-aortic balloon pump (IABP; OPS 8-83a.0*), percutaneous ventricular assist device (pVAD/Impella®, Abiomed, Danvers, MA, USA; OPS 8-83a.3*), and veno-arterial extracorporeal membrane oxygenation (V-A ECMO; OPS 8–852.3*).

### Statistical analysis

Descriptive patient data were analyzed using unpaired *t*-tests (assuming Gaussian distribution and similar standard deviations) for continuous variables. For categorical variables, Fisher’s exact test was used. Simple linear regression was used for the trendlines given in the figures, and the 95% confidence interval is displayed. For data on the monthly incidence of myocarditis, an adjustment for the number of days per month was computed using the formula: (“number of patients” / “days in month” / “months per year” × “days in year”). For the incidence of myocarditis per 100,000 inhabitants, the number of citizens of Germany for each year was obtained from DESTATIS. The number of COVID-19 cases per year was derived from the database of the Robert Koch Institute [24]. The significance level was set at a *p*-value < 0.05. Data are presented as absolute numbers and percentages for categorical variables, and continuous variables are presented as mean and standard deviation.

## Results

### General population

From January 1, 2007, to December 31, 2022 (i.e., 16 consecutive years), 88,159 patients were treated for myocarditis in hospital in Germany. Patients’ average age was 42 years, and 30% were female. Myocarditis was the main diagnosis of the hospital stay in 71% of these cases, and myocardial biopsies were performed in 7%. On average, hospitalizations lasted 8 days. One percent of patients received MCS. For details, see Table 1.

**Table 1:** Baseline characteristics and outcomes

	Whole cohort	COVID-19	Non-COVID-19	Sign
Column	1	2	3	2 vs 3
Years included	2007–2022	2020–2022	2020–2022	
Hospitalizations, *n* (%)	88,159 (100%)	1547 (1.75%)	16,229 (18.41%)	
Incidence per 100,000 inhabitants, *n*	6.70	0.62	6.47	
Myocarditis main diagnosis, *n* (%)	62,855 (71.30%)	756 (48.87%)	11,878 (73.19%)	< 0.0001
Biopsy performed, *n* (%)	6206 (7.04%)	46 (3.98%)^§^	1076 (6.63%)	0.0002
Age, mean ± SD [years]	42.48 ± 19.40	53.86 ± 23.27	42.68 ± 20.22	< 0.0001
Female gender, *n* (%)	26,487 (30.04%)	569 (36.78%)	4833 (29.78%)	< 0.0001
LOS, mean ± SD [days]	8.03 ± 11.56	12.34 ± 15.18	6.88 ± 8.88	< 0.0001
Ventilation > 48 h	2,885 (3.27%)	227 (14.67%)	432 (2.66%)	< 0.0001
CCI, mean ± SD []	0.80 ± 1.33	1.35 ± 1.78	0.87 ± 1.48	< 0.0001
Any MCS	1,158 (1.31%)	58 (3.75%)	219 (1.35%)	< 0.0001
V-A ECMO, *n* (%)	512 (0.58%)	25 (2.26%)^§^	110 (0.68%)	< 0.0001
PVAD, *n* (%)	376 (0.43%)	17 (1.47%)^§^	61 (0.59%)^§^	0.0018
IABP, *n* (%)	270 (0.31%)	0 (0%)	8 (0.08%)^§^	0.6075
COVID-19, *n* (%)	1547 (1.75%)	1547 (100%)	0 (0%)	< 0.0001
Hospital mortality, *n* (%)	2154 (2.44%)	194 (12.54%)	366 (2.26%)	< 0.0001

*LOS*, length of stay; *CCI*, Charlson Comorbidity Index; *MCS*, mechanical circulatory support; *V-A ECMO*, venoarterial extracorporeal membrane oxygenation; *PVAD*, percutaneous ventricular assist device; *IABP*, intra-aortic balloon pump

*P*-values are calculated between groups using either* t*-test or Fisher’s exact test as appropriate

^§^Some data points were redacted by DESTATIS due to low patient numbers

Characteristics and outcomes of patients hospitalized for myocarditis are displayed. Significance was calculated between the group of myocarditis with COVID-19 (from 2020 to 2022, i.e., column 2) and myocarditis without COVID-19 (from 2020 to 2022, i.e., column 3)

### Incidence of myocarditis

**Fig. 1:**
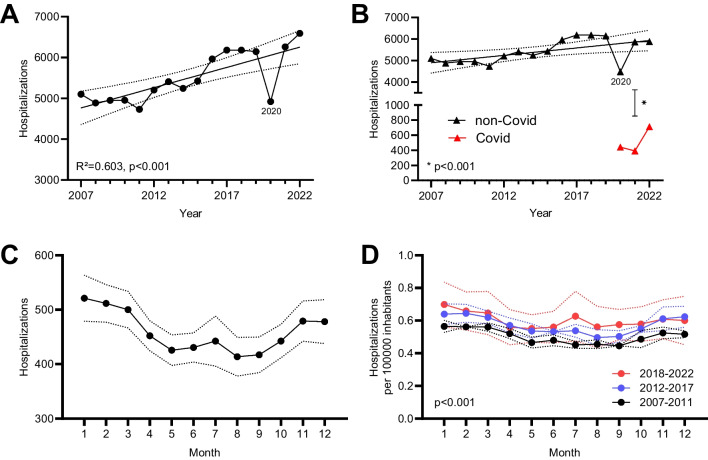
Myocarditis hospitalizations. Data adjusted for days per month and inhabitants of Germany. **A** Total hospitalizations with myocarditis per year. Annual hospitalization increased significantly from 2007 to 2022 with a pronounced dip in 2020. **B** Hospitalizations of myocarditis with or without COVID-19. Since the emergence of myocarditis with COVID-19 in 2020, it occurred significantly less frequently than myocarditis without COVID-19. **C** Hospitalizations with myocarditis per month. Peak levels of myocarditis occur in January. **D** Monthly hospitalizations for COVID-19 per months and period. Numbers of myocarditis were rising over the observed period (*p* < 0.001)

Hospitalizations of patients with myocarditis increased significantly over the observed period from 5100 in 2007 to 6593 in 2022 (*p* < 0.001 for trend) (see Fig. 1A). This trend persisted when excluding patients with COVID-19 (see Fig. 1B). Incidence per 100,000 inhabitants increased from 6.2 in 2007 to 7.8 in 2022 (*p* < 0.001 for trend, see supplemental Fig. 1C). There was a consistent increase in hospitalizations during the winter months compared to summer with the highest number of hospitalizations in January (527 hospitalizations, on average) and lowest in September (407, on average) (see supplemental Fig. 1A and B). Both trends, the increase over time and the higher rate in winter persisted when adjusting for days per month and the growing number of inhabitants of Germany (see Fig. 1C and D).

### Myocarditis mortality

**Fig. 2:**
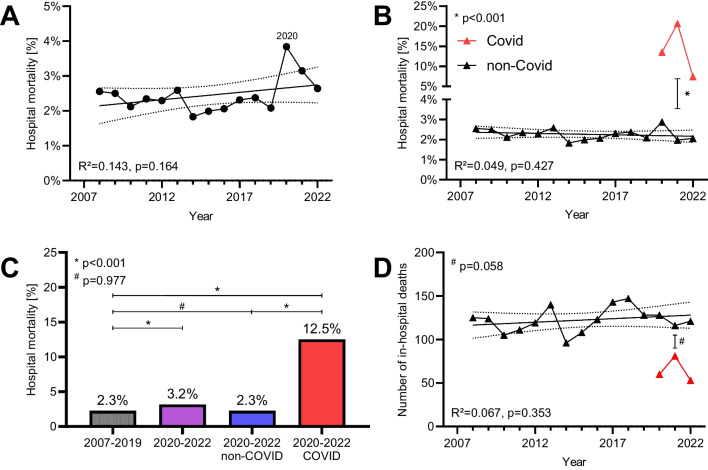
Myocarditis hospital mortality. **A** Mortality of hospitalizations with myocarditis per year. Hospital mortality was constantly around 2.5% between 2007 and 2022. **B** Hospital mortality with or without COVID-19. Myocarditis with COVID-19 showed significantly higher mortality than myocarditis without COVID-19. **C** Hospital mortality per period. The mortality of myocarditis without COVID-19 did not change after the emergence of COVID-19. **D** Number of in-hospital deaths per year. In-hospital deaths due to myocarditis did not change significantly over the investigated period (*p* = 0.353). Deaths with both myocarditis and COVID-19 were not significantly less than those due to myocarditis without COVID-19 in 2021 (*p* = 0.058)

During the study period, 2154 patients died during hospitalization with myocarditis, resulting in an in-hospital mortality rate of 2.44% (see Fig. 2A). The in-hospital mortality rate of patients hospitalized with myocarditis remained constant over time (*p* = 0.164 for trend), and total numbers remained constant over time, as shown in Fig. 2B–D.

### COVID-19 statistics

From 2020 to 2022, 37,392,371 COVID-19 cases were reported in Germany. During this period, 166,621 died after SARS-CoV2 being detected. COVID-19 mortality was highest in 2020 at 2.96%, while the highest absolute number of non-survivors was recorded at 65,966 in 2021. Hospitalizations rose every year to a maximum of 448,110 in 2022. For details, see Table 2.

**Table 2:** COVID-19 statistics in Germany according to DESTATIS and the Robert Koch Institut [24]

Year	2020	2021	2022	Total
Inhabitants in Germany, *n*	83,155,031	83,237,124	84,358,845	
Confirmed SARS-CoV-2 positive cases, *n*	1,782,948	5,439,638	30,169,785	37,392,371
Hospitalized with COVID-19, *n*	137,409	254,732	448,110	840,251
Hospitalizations with COVID-19 / 100,000 inhabitants,* n*	165.2	306.0	531.2	335.1
Hospitalizations with COVID-19 / 1000 positive cases, *n*	77.1	46.8	14.9	22.5
Non-survivors in SARS-CoV-2 positive cases, *n*	52,747	65,966	47,908	166,621
Mortality of SARS-CoV-2 positive cases, %	2.96%	1.21%	0.16%	0.45%
Hospitalizations with COVID-19 and myocarditis, *n*	443	392	712	1,547
Hospitalizations with myocarditis and COVID-19 / 100,000 inhabitants, *n*	0.53	0.47	0.84	0.62
Myocarditis / 1000 hospitalizations with COVID-19, *n*	3.2	1.5	1.6	1.8
Non-survivors in myocarditis and COVID-19, *n*	60	81	53	194
Mortality of hospitalizations with COVID-19 and myocarditis, %	13.54%	20.66%	7.44%	12.54%

Number of infections and case mortality are given for all patients and those hospitalized with myocarditis. Importantly, only myocarditis requiring hospitalization was included

### COVID-19 and myocarditis

Between 2020 and 2022, 1547 hospitalized patients were diagnosed with both COVID-19 and myocarditis, constituting 8.7% of all myocarditis cases during this period. Myocarditis cases with and without COVID-19 were significantly different in all baseline characteristics including the need for mechanical ventilation (*p* < 0.001). Significantly fewer patients with COVID-19 had myocarditis considered as the main diagnosis compared to patients without COVID-19 (48.9 vs. 73.2%, *p* < 0.001). Both V-A ECMO and PVAD were used significantly more often in patients with both, COVID-19 and myocarditis, compared to those with myocarditis alone (both *p* < 0.001) (see Table 1). Hospital mortality rate was significantly higher in patients with both, COVID-19 and myocarditis, compared to those with myocarditis alone (12.5% versus 2.3% respectively, *p* < 0.001) (see Fig. 2B and C).

## Discussion

Current data from DESTATIS show a significant rise in myocarditis hospitalizations in recent years up to 6593 cases in 2022. Incidence was 6.2/100,000 inhabitants in 2007 and 7.8/100,000 in 2022 following a seasonal pattern.

This is lower than the incidence in the USA averaging 10–14/100,000 [25]. Importantly, only 7% of patients in our dataset received cardiac biopsies considering the recommendations of the European Society of Cardiology [26]. The low utilization of myocardial biopsy is in accordance to recommendations in low-risk patients [27]. There are data suggesting a rise in MRI-facilitated myocarditis diagnosis in recent years [25]. Although we cannot exclude an under-reporting in our dataset, hospital mortality in the USA and in our dataset is similar [28]. This makes it unlikely that severe cases are missed in the current research.

Influenza being a known infectious agent in myocarditis [13] strongly corresponds to the highest rates of myocarditis hospitalizations in January and February during the flu season in the northern hemisphere. A causal link between flu and myocarditis hospitalizations however cannot be drawn from our dataset, while the gender gap of men suffering from myocarditis significantly more often than women is well established [29]. Sex hormones may play a role in this pronounced gender gap [30].

Since 2020, a subset of about 1500 patients was hospitalized with both, COVID-19 and myocarditis. The incidence of hospitalizations with myocarditis and COVID-19 in our dataset was 180/100,000 hospitalizations with COVID-19, comparable to the rate of 146–240/100,000 individuals seen in other registries [19, 31]. The low incidence of myocarditis hospitalizations in 2020 might be explained by an underutilization of the healthcare system during the COVID-19 pandemic [32–35]. Since our first analysis of myocarditis in 2020 [20], SARS-CoV-2 variants developed from Alpha to Omicron [6, 36–39]. Dominant variants switched regularly [40] as did outcome parameters of COVID-19 including case fatality rate (highest 2020), mortality (highest 2021), and infections (highest 2022). If and how virus variants influence myocarditis is unknown.

Patients hospitalized with both, COVID-19 and myocarditis, had a significantly higher hospital mortality rate of 12.5% compared to 2.3% in hospitalizations with myocarditis without COVID-19. As the combination of hospitalization for myocarditis and COVID-19 without respiratory failure seems to be a rare occurrence [41–43], it is tempting to speculate on cofactors such as acute respiratory distress syndrome (ARDS) being the main driver of mortality [44, 45] in myocarditis with COVID-19. ARDS does not only impair gas exchange, but also increases right ventricular afterload [46, 47], which might be especially detrimental in case of myocarditis. Since the cohorts of myocarditis with and without COVID-19 also differed significantly in several important characteristics including age and comorbidities, ARDS however might not be the only driver of mortality in myocarditis with COVID-19.

## Limitations

Data presented derives from the Federal Statistical Office of Germany (DESTATIS). Data on non-reimbursement-relevant comorbidities is likely to be underreported and therefore not included in the current investigation. Data from clinical investigations like transthoracic echocardiogram, MRI, myocardial biopsies, or laboratory tests are not included in the DESTATIS dataset. The exact cause of death cannot be derived from the dataset. Also, the diagnosis of myocarditis could not be reviewed independently.

## Conclusion

Overall numbers of hospitalization for myocarditis rose from 2007 to 2022, while mortality remained low. A minority of patients were hospitalized with both, COVID-19 and myocarditis. This distinct subgroup had different patient characteristics and a higher hospital mortality rate.

## Supplementary Information

Below is the link to the electronic supplementary material.

Supplementary file1 Supplementary figure 1: Myocarditis hospitalizations. Data not adjusted for days per month and inhabitants of Germany. A: Hospitalizations with myocarditis per month. Peak levels of myocarditis occur in January. B: Monthly hospitalizations for COVID-19 per months and period. Numbers of myocarditis were rising over the observed period (p<0.001). C: Hospitalizations per 100,000 inhabitants and year. Numbers were rising significantly in the investigated time frame (p<0.001).(DOCX 221 KB)

## Data Availability

All data is publicly available from DESTATIS and the RKI.
