# Supplementary material for: Myocarditis incidence and hospital mortality from 2007 to 2022: insights from a nationwide registry
Source: Clin Res Cardiol. 2024 Aug 26;114(9):1156–63. doi: 10.1007/s00392-024-02494-3 (PMC12408657; doi:10.1007/s00392-024-02494-3)
Supplement: Supplementary file 1 — Supplementary file1 Supplementary figure 1: Myocarditis hospitalizations. Data not adjusted for days per month and inhabitants of Germany. A: Hospitalizations with myocarditis per month. Peak levels of myocarditis occur in January. B: Monthly hospitalizations for COVID-19 per months and period. Numbers of myocarditis were rising over the observed period (p<0.001). C: Hospitalizations per 100,000 inhabitants and year. Numbers were rising significantly in the investigated time frame (p<0.001).(DOCX 221 KB) [file 392_2024_2494_MOESM1_ESM.docx]

# Supplementary material

**Supplementary figure 1: Myocarditis hospitalizations.** Data not adjusted for days per month and inhabitants of Germany. A: Hospitalizations with myocarditis per month. Peak levels of myocarditis occur in January. B: Monthly hospitalizations for COVID-19 per months and period. Numbers of myocarditis were rising over the observed period (p<0.001). C: Hospitalizations per 100,000 inhabitants and year. Numbers were rising significantly in the investigated time frame (p<0.001).
